# Notch strengthening or weakening governed by transition of shear failure to normal mode fracture

**DOI:** 10.1038/srep10537

**Published:** 2015-05-29

**Authors:** Xianqi Lei, Congling Li, Xinghua Shi, Xianghong Xu, Yujie Wei

**Affiliations:** 1 LNM, Institute of Mechanics, Chinese Academy of Sciences, Beijing 100190, P.R. China; 2 School of Civil Engineering, Luoyang Institute of Science and Technology, Luoyang, Henan province, 471023, P.R. China

## Abstract

It is generally observed that the existence of geometrical discontinuity like notches in materials will lead to strength weakening, as a resultant of local stress concentration. By comparing the influence of notches to the strength of three typical materials, aluminum alloys with intermediate tensile ductility, metallic glasses with no tensile ductility, and brittle ceramics, we observed strengthening in aluminum alloys and metallic glasses: Tensile strength of the net section in circumferentially notched cylinders increases with the constraint quantified by the ratio of notch depth over notch root radius; in contrast, the ceramic exhibit notch weakening. The strengthening in the former two is due to resultant deformation transition: Shear failure occurs in intact samples while samples with deep notches break in normal mode fracture. No such deformation transition was observed in the ceramic, and stress concentration leads to its notch weakening. The experimental results are confirmed by theoretical analyses and numerical simulation. The results reported here suggest that the conventional criterion to use brittleness and/or ductility to differentiate notch strengthening or weakening is not physically sound. Notch strengthening or weakening relies on the existence of failure mode transition and materials exhibiting shear failure while subjected to tension will notch strengthen.

Circumferentially notched bars are known to induce stress triaxiality: Shear deformation in the neck of such samples is confined by the shoulders, which gives rise to high hydrostatic tension^1^,^2^. The influence of hydrostatic tension by shoulder constraint on the strength of materials is twofold: Hertzberg^3^ observed that for high-strength steel, its strength decreases with increasing notch depth; however, the strength of low carbon steel increases as notches becomes deeper. Hertzberg’s observation leaded to the conclusion that *brittle materials will notch weaken and highly ductile materials will notch strengthen*^3^. Later on experiments to explore normal mode fracture in bulk metallic glasses (BMG) showed that the hydrostatic stress plays an important role on flow localization in notched BMG samples which could trigger cavitation failure^4^. The authors also reported that the failure stress in the notched region decreased with increasing triaxiality. Recent observation by Wang *et al.*^5^ revealed that a notch BMG sample has actually higher strength than that of an intact sample. The tests conducted by Varadarajan and Lewandowski^6^, with superimposed hydrostatic pressure to notched BMG samples, showed that the strength of those samples at fracture was nearly unaffected. So far, there is no consensus about the influence of notches to the strength of materials: While materials without tensile ductility like BMGs notch strengthens^5^, there also exists observation that hydrostatic pressure has negligible impact to the fracture strength^6^ of BMGs. In addition, the notch strengthening observed in brittle BMGs^5^ is in confliction with the statement that brittle materials will notch weaken^3^. Those inconsistencies indicate that further understanding about the role of hydrostatic stress (resulted from notches) on the strength of materials remains unclear. It hence calls for more systematic and well controlled experiments to shed light on how notches may influence the strength of materials and the behind mechanisms responsible for the observed phenomena.

## Results

### Characterization on notch strengthening and weakening

**Figure 1:**
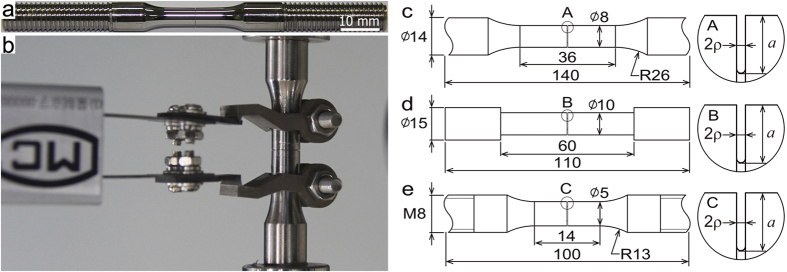
Mechanical characterization of circumferentially notched samples. (**a**) A typical notched BMG sample. (**b**) Setup of the test to measure the displacement of the notched section, and the gauge length of the extensometer is 7 mm. (**c**)–(**e**) Critical dimensions of Al6061, ceramics Al_2_O_3_ and Zr_41_Ti_14_Cu_12.5_Ni_10_Be_22.5_ (Vitreloy 1) samples, with notch depth 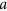 and notch radius 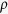.

**Figure 2:**
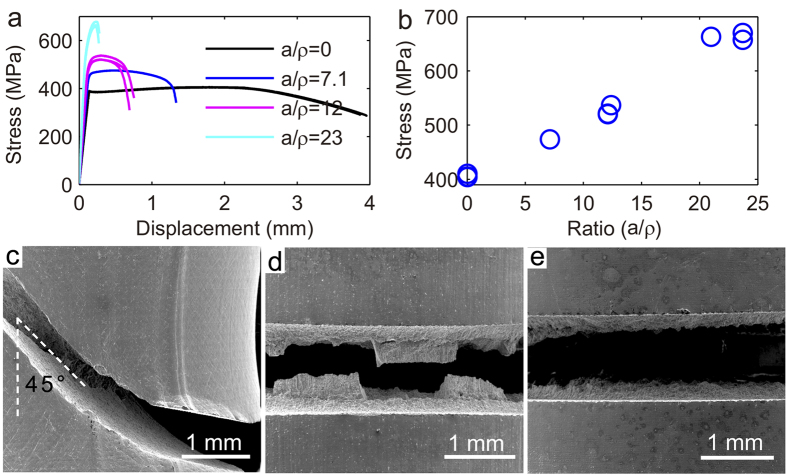
Notch-strengthening in Al T6061. (**a**) Stress-displacement curves of samples with different constraint. (**b**) Peak strength as a function of constraint 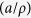. (**c**) The sample without notch 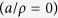 has a fracture angle of 45 degrees. (**d**) and (**e**) Samples with 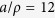 and 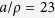, respectively, have fracture angles of 90 degrees.

To address the questions raised above, we investigated the influence of notches to the strength of materials of distinct mechanical properties: Polycrystalline aluminum T6061 which has intermediate tensile ductility, Zr_41_Ti_14_Cu_12.5_Ni_10_Be_22.5_ (Vitreloy 1) metallic glasses which has no tensile ductility, and brittle ceramic Al_2_O_3_. We first explored the dependence of strength on the constraint defined as the ratio of notch depth over notch root radius (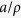) in aluminum T6061. Detailed information about sample preparation and characterization can be found in Fig. 1 and the Method section. The mechanical behavior of circumferentially notched Al T6061 is shown in Fig. 2. From the stress-displacement curves (Fig. 2a) and the peak strength as a function of the constraint 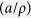 shown in Fig. 2b, we see significant strengthen enhancement when 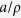 increases: The yielding strength of an Al T6061 bar is about 390 MPa, in contrast to the yield strength of 680 MPa in the circumferentially notched bar with 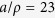. The intact sample (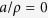) has a fracture angle of about 45^o^ (Fig. 2c). While increasing the constraint to 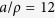 or 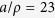, we see respectively from Fig. 2d or Fig. 2e that the fracture angle becomes 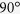, demonstrating the transition from mode II type shear failure to normal mode I fracture as 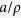 increases. Generally, ductile metals like Al T6061 exhibit pressure-independent behavior if they fail in shear. In such circumstance, the strength reflects the shear resistance of materials even they are subjected to tension. In notched Al T6061, however, the shoulder constraint triggers normal (mode I) fracture instead of shear failure.

**Figure 3:**
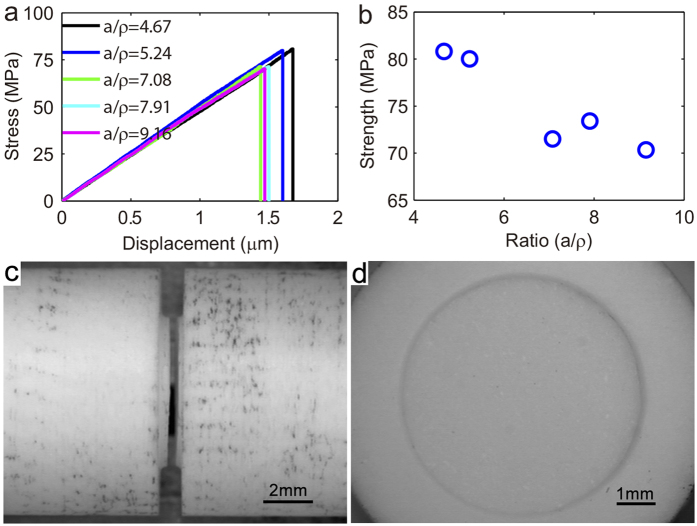
Notch weakening in brittle ceramic Al_2_O_3_. (**a**) Stress-displacement curves of samples with different constraint. (**b**) Peak strength as a function of constraint 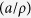. (c-d) Side view and top view of the fractured samples, respectively, to show mode I fracture at different 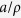, suggesting notch weakening in brittle ceramics is governed by stress-concentration.

**Figure 4:**
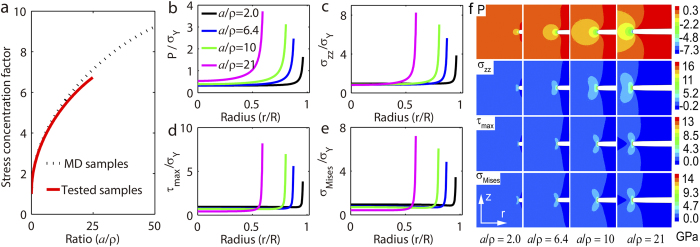
Stress concentration and stress evolution in notch necks in elastic media. (**a**) Stress concentration factor *K* for axial stress as a function of the constraint 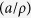: Predictions from the Neuber’s trigonometric formula (eqns. 1 and 2). The concentration factors for tested BMG samples and also simulated MD samples are shown. A monotonic increasing of stress concentration factors is seen in elastic media. (**b**) to (**e**) stress along the radial direction in different geometrical constraint from finite element simulations for elastic media: (**b**) the hydrostatic pressure; (**c**) the axial normal stress; (**d**) the maximum shear stress; and (**e**) the von Mises stress. (**f**) Contours from finite element simulations, from top to bottom, show in turn the hydrostatic pressure, the axial tension, the maximum shear stress, and the von Mises stress in samples with different 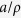.

The mechanical behavior of the notched ceramic Al_2_O_3_, however, is different from that of Al T6061. We observed strength weakening from Fig. 3a,b. Fracture in the ceramic is dominantly mode I type, regardless the variation in 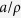; i.e., no deformation transition presents (Fig. 3c,d) in the ceramic. The notch weakening in ceramic is consistent with the general viewpoint of stress concentration leads to strength weakening^3^: Stress concentration increases as a resultant of deeper notching. If we consider the notch as a crack, Griffith strength theory^7^ predicts that the failures stress 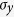 of a cracked sample follows 
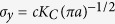
 for 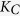 being the fracture toughness and 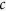 being a geometrical factor. The Griffith theory predicts that the strength of notched samples decrease when the notch depth increases. If we further consider the influence of notch root radius, it is known that the stress concentration factor 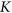 for cylinders having finite diameter and finite notch depth can be obtained by using the Neuber’s trigonometric formula^1^
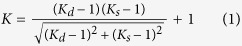
where 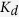 and 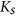 are respectively the exact stress concentration factors of deep notches and shallow notches in an infinitely large cylinder. When bars with circumferential notches are subjected to uniaxial tension, we have
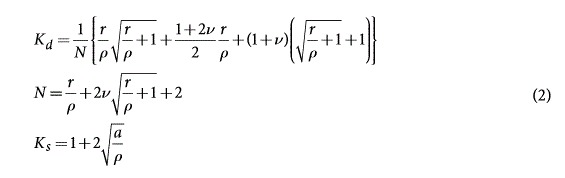
where 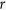 is the radius of the notch neck and 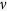 is the Poisson’s ratio of the material. The stress concentration factor 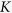 increases monotonically with notch depth when the notch root radius is a constant, as clearly seen in Fig. 4a. The theoretical prediction that the higher stress concentration in deeper notched samples is consistent with our finite element simulations for elastic media shown in Fig. 4. From Fig. 4b to 4e, we show stress evolution in notch necks while the sample deforms elastically. The stress components including the hydrostatic pressure (Fig. 4b), the normal stress (Fig. 4c), the maximum shear stress (Fig. 4d), and the von Mises stress (Fig. 4e) exhibit severer level of stress concentration when notches become deeper. Fig. 4f, from top to bottom, shows in turn the contours of hydrostatic pressure, normal stress, maximum shear stress, and von Mises stress in samples with different 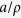 when the material deforms elastically. Based on the above theoretical analysis, we see that increasing 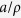 should then degrade the strength of the notch neck if the von Mises or maximum shear failure criterion is applicable to the elastic material. That is probably the situation for the notched ceramic before it fails.[Table t1][Table t2]

**Figure 5:**
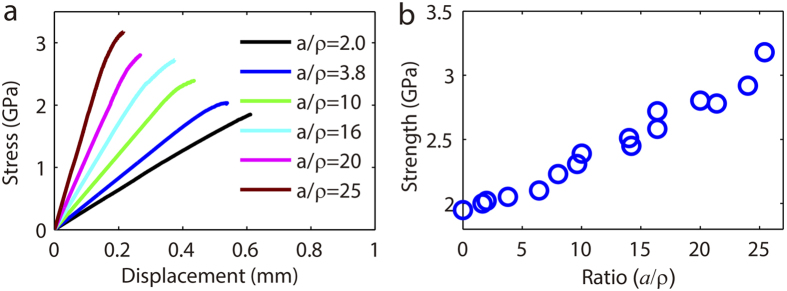
Mechanical characterization of circumferentially notched BMG Vitreloy 1 samples. (**a**) Stress-displacement curves of notched samples with different constraint 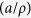. (**b**) The strength of notched samples as a function of constraint 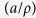.

Given BMG Vitreloy 1 only has about 2% elastic strain before failure^8^,^9^, it has very limited deformation capacity and is regarded brittle. In this sense, we expect notch weakening in BMG Vitreloy 1. We show in Fig. 5a the stress-displacement curves of the notched BMG Vitreloy 1 with different geometrical constraints. The strength as a function of constraint 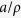 is shown in in Fig. 5b. There is significant notch strengthening in Vitreloy 1. It is counter-intuitive as notch strengthening in BMGs contradicts to the general observation that brittle materials will notch weaken^3^. The increase of tensile strength with increasing 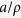 is significant: The tensile strength of an intact BMG Vitreloy 1 sample is about 1.9 GPa while that of the deep notched sample with 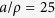 reaches 3.2 GPa. A total of 18 samples were tested and their corresponding dimensions are tabulated in Table 3.

**Figure 6:**
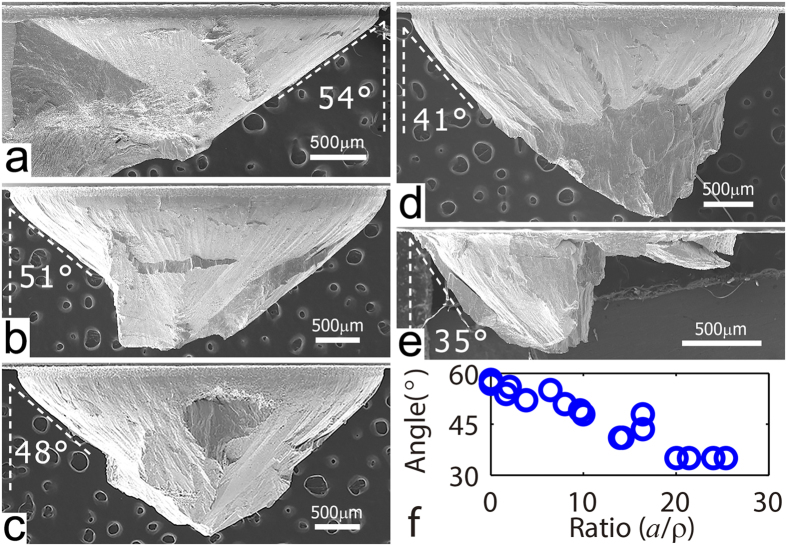
Macroscopic fractography of notched samples after tensile failure. (**a**) to (**e**) SEM images to show final fractured samples with different 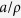 and their shear angles. (**a**) 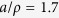. (**b**) 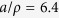. (**c**) 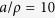. (**d**) 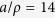. (**e**)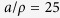. (**f**) The angle between the loading direction and the shearing surfaces as a function of constraint 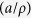.

The notch strengthening in BMGs does not seem to be caused by the general ‘*smaller being stronger*’ observation in crystalline metals. Indeed, BMGs do not show apparent size-effect in samples at the micron scale or bigger^10^,^11^,^12^,^13^,^14^. Given the sizes of samples are far greater than microsize, the observed notch strengthening is not a cause of the reduced neck size. In order to shed light on the strengthening behavior shown in Fig. 5, we examined the macroscopic fractographies of those notched samples. Figs. 6a to 6e, in turn, show the fracture surfaces of samples with constraints 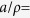
1.7, 6.4, 10, 14, and 25. It is seen that the fractured surfaces form a conic tip, and the shear angle increases with increasing constraints. At the low level of constraint 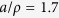, we have 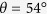. The shear angle is close to that of an intact sample after tensile failure. For the latter case, 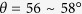. While 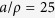, we see that 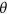 approaches 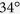 (see Fig. 6e). The angle may become 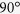 where the fracture plane is perpendicular to the loading axis due to the transition from shear band dominated mode II failure to normal mode I fracture.

### Notch effect: Molecular dynamics simulations

**Figure 7:**
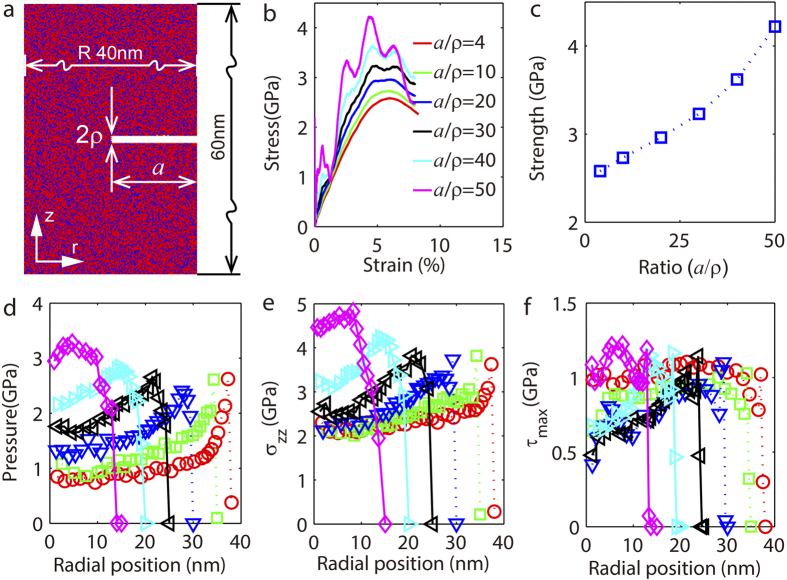
Molecular dynamics simulations to show mechanical behavior of circumferentially notched bars under tension. (**a**) The dimensions of a circumferentially notched Ni_30_Zr_70_ sample used in molecular dynamics simulations (Ni, blue; Zr, red). The notch root radius 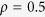 nm and the notch depth a varies from 2 nm to 25 nm: (**b**) stress-strain curves; (**c**) strength as a function of constraints. (**d**) to (**f**) Evolution of stress components along the radial different for several 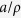 ratios: (**d**) the hydrostatic tension; (**e**) the axial stress; and (**f**) the maximum shear stress.

To understand the atomistic deformation mechanisms accounting for the anomalous strengthening in metallic glasses with circumferential notches, we conducted molecular dynamics (MD) simulations using the binary amorphous metallic glass Ni_30_Zr_70_. While being much simpler in composition, this BMG owes mechanical properties close to BMG Vitreloy 1. It enables us to explore the general physics behind the strengthening effect. The axial-symmetrical projection of a circumferentially notched Ni_30_Zr_70_ sample used in our molecular dynamics simulations (Ni, blue; Zr, red) is shown in Fig. 7a. The notch tip radius is fixed to 0.5 nm but its depth varies from 2 nm to 25 nm. More detailed information about our MD simulations can be seen the Method section.

**Figure 8:**
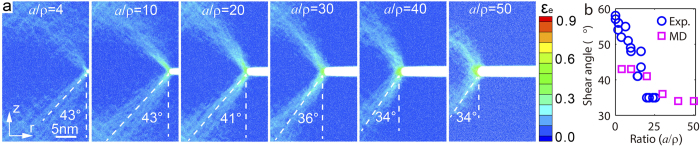
Molecular dynamics simulations to show mechanical behavior of circumferentially notched bars under tension. (**a**) Strain contours in samples with different 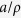 ratios, where the angle between the shear direction and the vertical loading axis is marked for each simulation. (**b**) Slip direction versus 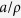 ratios from both experimental measurement (Vitreloy 1) and MD simulations (Ni_30_Zr_70_).

While strain rate issue in molecular dynamics (MD) simulations is prevailing^15^,^16^, we note that we compared all MD simulations at the same strain rate, and checked the strength of those samples subjected to the same boundary conditions. In that sense, our MD simulations are self-consistent. Their relative strengths of samples with different notch depth can be used to reveal the deformation mechanisms accounting for notch effects in metallic glasses. Fig. 7b shows the curves of stress-strain for samples with notch depth of 2 nm (red), 5 nm (green), 10 nm (blue), 15 nm (black), 20 nm (cyan) and 25 nm (magenta). Here the strain is defined as ratio of the elongation of the sample to its initial length, and the stress is the true stress in the neck. The strengths of samples with different constraints 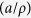 are shown in Fig. 7c. We see that the strengths increase substantially as notches become deeper, which supports the experimental observations shown in Fig. 5. We also abstract the hydrostatic tension 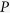, the normal stress along the loading axis 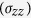, and the maximum shear stress 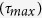 in the neck of a sample. Figs. 7d to 7f show respectively those stress components along the radial direction. Three significant features are seen here: Firstly, both the hydrostatic tension and the normal stress increase dramatically with increasing 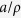, in particular near the central of the circular neck section. Secondly, the peak hydrostatic stress 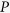 and the maximum normal stress 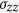 occur at locations with some distance away from the notch tip, and they also increase mildly when 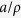 increases. Lastly, the maximum shear stress does not show clear dependence on 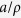. These observations suggest that the peak hydrostatic tension and the maximum normal stress 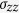 are responsible for the increasing strength with constraint 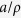 (shown in Fig. 7c). They account for mode I fracture rather than shear failure in deep notched samples. Consequentially, the transition in failure mode gives rise to strengthening shown in stress-strain curves (Fig. 7b). The mechanisms revealed by our atomistic scale simulations in general support the observations seen in Fig. 2 and 5 in macroscopic samples. We show in Fig. 8a the equivalent shearing strain at the atomistic level in the notched region. The conic shearing surface is seen, which agrees with experimental observations shown in Fig. 6. The shear angle versus 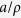 shown in Fig. 8b agrees well with experimental measurement given in Fig. 6f.

### Notch effect: Finite element modeling

**Figure 9:**
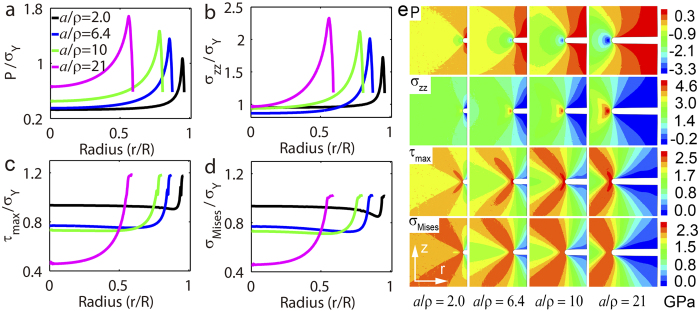
Stress evolution from finite element simulations in notch necks in elastic-plastic media. (**a**) to (**d**) Stress along the radial direction in different geometrical constraint: (**a**) hydrostatic pressure; (**b**) axial tension; (**c**) the maximum shear stress; (**d**) von Mises stress. (**e**) Contours from top to bottom, in turn, show the hydrostatic pressure, the axial tension, the maximum shear stress, and the von Mises stress in samples with different 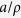.

We also performed finite element simulations in the notched samples for elastic- plastic media. For simplicity but without loss of physics, we assume the modeled material deforms elastic-plastically. We use a yielding strength 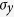 = 1950 MPa, which equals to the strength of BMG Vitreloy 1. In contrast to the high strain rates and the small samples used in MD simulations, sample sizes and loading loads used in finite element simulations are accessible in laboratory. Similar to the information shown in Fig. 7, we show in Figs. 9a to 9d, respectively, the pressure 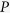, the normal stress 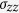, the von Mises stress 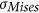, and the maximum shear stress 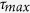 along the radial direction in the neck section. It is consistently seen that both the hydrostatic tension and the normal stress increase dramatically with increasing 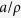. More importantly, both the Mises stress and the maximum shear stress become smaller with increasing 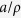, which excludes their role to account for the strengthening effects revealed by experiments (Figs. 2 and 5) and MD simulations (Fig. 7). Those observations from above simulations also lead to the conclusion that the peak hydrostatic tension and/or the maximum normal stress 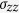 play the governing role for the increasing strength with 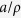 in Al T6061 and BMGs.

## Discussion

By exploring the mechanical response of three distinct materials – Al T6061, BMG Vitreloy 1, and ceramic Al_2_O_3_, we obtain the following three conclusions: (1) Al T6061 and BMGs exhibit notch-strengthening with increasing constraints, and the normal stress eventually reaches cohesive strength of the materials and leads to mode I fracture. (2) Notch strengthening in Al T6061 and BMGs is a resultant of deformation transition from shear failure to mode I fracture. The former is associated with shear strength yet the latter is governed by the cohesive strength of materials^17^. (3) Notch strengthening in both BMG Vitreloy 1 with only 2% tensile strain and ductile aluminum alloys suggests that the materials exhibiting shear failure in tension will notch strengthen, which overthrows the conventional viewpoint that brittle materials will notch weaken and highly ductile materials will notch strengthen. The strengthening effect observed here paves the way to validate and calibrate parameters in yielding or failure criteria for metals. More physically sound yielding or failure criteria could be developed as the competition of the two failure mechanisms exhibited in one type of experiment allows us to probe the shearing strength and mode I failure strength. In engineering practice, circumferential notches or groove are structural characteristic broadly used in machine elements such as turbine rotors blade rows and a variety of shafts. The physical mechanisms responsible for notch strengthening/weakening, as reported here, could better our design for safety factors of such commonly seen structures.

## Methods

### Sample preparation

Zr_41_Ti_14_Cu_12.5_Ni_10_Be_22.5_ (Vitreloy 1) metallic glasses are made in a water-cooled arc-melting hearth under a titanium-gathered argon atmosphere. Elemental metals (>99.9% purity) are used to form the master alloy and suction-casted in 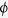
8 mm × 100 mm cylinders. A typical sample with a circumferential notch in the middle is shown in Fig. 1a. The notch is prepared by electrical discharge machining (EDM). To make circumferential notches smooth and to remove possible damage induced by EDM, we further cleaned notched samples by electro-polishing. The Aluminum 6061 and ceramic Al_2_O_3_ were commercially bought. The layout and the critical dimensions (in unit of mm) of samples of Al 6061, ceramic Al_2_O_3_, and metallic glass Zr_41_Ti_14_Cu_12.5_Ni_10_Be_22.5_ (Vitreloy 1) are presented, in turn, in Fig. 1c to Fig. 1e. The reader may refer to Tables 1, 2, 3 for the detailed dimensions of the three different types of samples, respectively.

### Mechanical testing

A servo-hydraulic MTS 810 test system is used to do the tension test. Experimental setup to ensure an accurate measure of displacement across a notch is shown in Fig. 1b. A displacement gauge with a resolution of 0.2μm was attached to a specimen. Universal joints were employed to eliminate moment influence during tensile loading because of possible misalignment. We used a loading rate of 0.5 mm/min (corresponding to a strain rate of 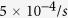). We define the strength of the neck as the maximum force over the area of the minimum cross-section of the neck.

### SEM characterization

FEI Quanta 200 scanning electron microscope (SEM) from Fédération Equestre Internationale was employed for microstructure characterization.

### Molecular dynamics simulations

The MD simulations were conducted using LAMMPS^18^, which is a widely used open source code. Atomic interactions were modeled by embedded atom method potentials with parameters given by Mendelev *et al.*^19^. Glass samples consisting of a randomly substituted solid solution (satisfying the ratio to ensure Ni_30_Zr_70_ in a face-centred cubic lattice are used in a melting-and-quenching simulation, during which the system temperature was raised gradually from 0 to 2100 K and was then cooled down to 300 K. The cooling rate was set at 18 K/ns. The final BMG sample with dimensions of 80 nm × 80 nm × 60 nm is used, and the samples contain about 20,000,000 atoms. A circumferential notch is introduced in the body of BMG with notch width 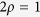 nm. The final notch neck is a circle whose radius depends on the notch depth 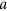. Several samples with different notch depths were prepared, including *a *=* *2, 5, 10, 15, 20 and 25 nm. The axial-symmetrical projection of a circumferentially notched Ni_30_Zr_70_ sample used in our molecular dynamics simulations (Ni, blue; Zr, red) is shown in Fig. 7a. The notch root radius 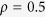 nm, and the notch depth 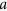 varies from 2 nm to 25 nm. Table 4 supplies the information about the geometrical details of all samples used for MD simulations. The time step for integration was chosen to be 1 fs. In all simulations the periodic boundary condition (PBC) was applied in all directions. The MG samples were then loaded under uniaxial tension at the strain rate of 10^8^ s^−1^. The equivalent strain was calculated via the method provided by Falk^20^.

### Finite element simulations

We model the stress fields in the notched samples for both elastic media and elastic-perfectly plastic media. The Young’s modulus 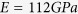 and Poisson’s ratio 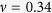. The geometry details of samples used for FEM simulation is tabulated in Table 5.

## Additional Information

**How to cite this article**: Lei, X. *et al.* Notch strengthening or weakening governed by transition of shear failure to normal mode fracture. *Sci. Rep.* **5**, 10537; doi: 10.1038/srep10537 (2015).

## Figures and Tables

**Table 1 t1:** Detailed information about circumferentially notched aluminum T6061. The dimensions, the maximum strength, and their corresponding fracture angles (the angle between the fracture surface and the loading axis) of typical samples are shown.

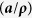	**2R(mm)**	**2r(mm)**	**Depth** 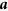 **(μm)**	**Width** 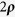 **(μm)**	**Strength (MPa)**	**fracture angle**
0	8.16	8.16	0	0	405	45°
7.1	8.15	7.1 5	500	140	474	90°
12.1	8.15	6.45	850	140	521	90°
12.4	8.16	6.43	865	140	537	90°
23.7	8.14	4.82	1660	140	670	90°

**Table 2 t2:** Detailed information about circumferentially notched ceramic Al_2_O_3_. The dimensions, the maximum strength, and corresponding fracture angles (the angle between the fracture surface and the loading axis) of typical samples are shown.

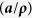	**2R(mm)**	**2r(mm)**	**Depth** 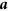 **(μm)**	**Width** 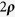 **(μm)**	**Strength (MPa)**	**fracture angle**
4.67	10.00	8.04	980	420	80.8	90°
5.24	10.00	7.80	1100	420	80.0	90°
7.08	10.00	6.60	1700	480	71.5	90°
7.91	10.02	6.45	1780	450	73.4	90°
9.16	10.00	6.06	1970	430	70.3	90°

**Table 3:** Detailed information about circumferentially notched BMG Vitreloy 1. The dimensions, the maximum strength, and their corresponding fracture angles (the angle between the fracture surface and the loading axis) of all samples are shown.

**Constraint** 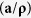	**2R (mm)**	**2r (mm)**	**Depth** 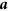 **(μm)**	**Width** 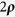 **(μm)**	**Strength (GPa)**	**fracture angle**
0	4.29	4.29	0	N/A	1.98	57°
1.66	4.00	3.82	100	120	1.99	56°
2.00	4.30	4.05	110	110	2.02	56°
3.80	4.26	3.87	192	110	2.05	52°
6.40	5.15	4.51	320	100	2.10	55°
8.00	5.16	4.38	400	100	2.23	51°
9.64	5.16	4.08	502	109	2.31	49°
10.00	5.13	4.13	500	100	2.39	47°
14.00	5.12	3.72	700	100	2.51	41°
14.20	5.14	3.72	710	100	2.45	41°
16.40	4.54	2.90	820	100	2.72	48°
16.40	5.10	3.46	795	100	2.58	44°
20.00	5.15	3.06	1070	106	2.80	35°
21.40	5.16	3.02	1070	100	2.78	35°
24.00	5.18	2.78	1200	100	2.92	35°
25.40	5.10	2.06	1520	120	3.18	35°

**Table 4:** Notch depth and system size for BMG samples (Ni_30_Zr_70_) used in MD simulations. A constant notch root radius 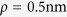 is used for all samples.

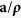	**2R (nm)**	**2r (nm)**	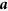 **(nm)**	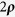 **(nm)**	**Atoms**	**Strength (GPa)**
4	80	76	2	1	19592188	2.58
10	80	70	5	1	19557037	2.73
20	80	60	10	1	19504930	2.92
30	80	50	15	1	19460793	3.23
40	80	40	20	1	19424675	3.62
50	80	30	25	1	19396593	4.22

**Table 5:** Notch depth and system size for samples used in finite element simulations.

**a/ρ**	**2R (mm)**	**2r (mm)**	**a(μm)**	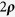 **(μm)**	**Maximum Load(KN)**
2.0	5.15	4.95	100	100	38.8
6.4	5.15	4.51	320	100	33.3
10	5.15	4.15	500	100	32.0
21	5.15	3.06	1050	100	20.6
